# Frequency of needle stick injuries among healthcare providers during large-scale SARS-CoV-2 vaccination brigades

**DOI:** 10.3389/fpubh.2023.1084812

**Published:** 2023-02-14

**Authors:** Jorge Daniel Moran-González, Magaly Padilla-Orozco, Abel Guzman-Lopez, Hilda Cristina Ochoa-Bayona, Adrian Camacho-Ortiz

**Affiliations:** Universidad Autóma de Nuevo León, Hospital Universitario Dr. José Eleuterio González, Monterrey, Mexico

**Keywords:** needle stick and sharp injuries, COVID-19, vaccination, brigade, healthcare provider

## Abstract

The frequency of needle stick-related accidents in large-scale vaccination brigades during the COVID-19 pandemic is unknown. We determined the incidence of needle stick injuries (NIs) from the SARS-CoV-2 vaccination brigades in the Monterrey metropolitan area. We calculated the rate of NI by 100,000 doses administered from a registry of over 4 million doses.

## Introduction

According to the World Health Organization, more than two million occupational exposures to sharp injuries occur among 35 million healthcare providers (HPs) annually (1, 2). Needle stick injuries among nurses were associated with three factors: nurses' sense of urgency, variable shift work, and lower skill level related to the years of experience, academic degree, and younger age (3).

The increased volume of vaccination campaigns during a pandemic scenario resulted in a considerable increase in needle stick injuries during mass vaccination programs compared to routine vaccination during the 2009 influenza A (H1N1) pandemic. Contributing factors such as a higher rate of vaccinations administered by HP, non-hospital settings, and a lack of formal training were considered (4).

The incidence of these needle stick-related accidents in large-scale vaccination brigades during the COVID-19 pandemic is unknown.

We sought to determine the incidence of needle stick injuries (NIs) in the SARS-CoV-2 vaccination brigades in the Monterrey metropolitan area. For this purpose, a cross-sectional analysis was carried out on a cohort of HP where every occupational exposure by puncture and/or splash exposure (SE) are recorded by all HP who participated in vaccination brigades. Variables were expressed as means and percentages, Wilcoxon rank test and chi-square test were employed. We used SPSS version 20.0 for analyses.

During this study period, 872 brigades encompassed 49,879 HPs with a total of 4,144,052 vaccine doses applied. Of these, 91 NI were reported, and 3 SEs from a total of 19,950 HPs actively participated in applying the vaccines with a constant shift rotation. We calculated the rate by 100,000 doses administered and classified the accidents by the age of the HP, the department where the HP was assigned, and the stage of the vaccination process in which the accident occurred. HPs, who did not apply vaccines, were not considered for analysis.

Only 6 NIs (6.3%) occurred in healthcare workers compared to 88 accidents (93.6%) that occurred in medical personnel in training (*p* = 0.0001). The age range of affected HP was between 19 and 21 years (42.5%), of which 59 cases (57.44%) were nursing students. Most of the accidents (27.5%) occurred during the puncture process. All NIs/SEs were notified and referred to the epidemiology department for evaluation and follow-up. Every HP was scheduled for sequential clinical and serologic follow-up during the initial 6-month period after the accident. However, the average follow-up was 80.6 days (0–272 days). All personnel who suffered an NSI/SE have sequential serologic tests and no seroconversion was detected.

**Figure 1:**
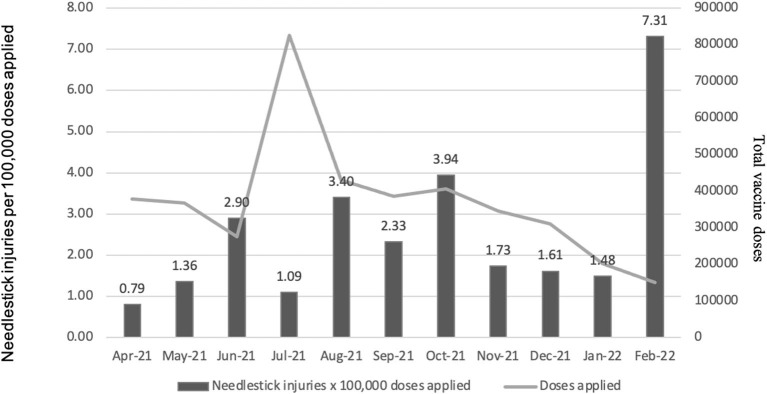
Rate of needle stick injuries × 100,000 doses among healthcare providers.

The average rate of accidents was 2.79 per 100,000 doses (range 0.79–7.31), 2.54 NI per 100,000 doses (range 0.79–7.31) (Figure 1), 4.184 SE per 100,000 doses (range 2.59–5.78), and 1.24 per 100,000 doses (range 0.26–7.31) in medical personnel in training. There was a low correlation between the monthly number of doses applied and the number of accidents (*Kappa* 0.58). Continuous medical personnel training is a key element in preventing occupational accidents (5, 6). Factors such as a lack of formal training and the large volume of people who require a vaccine on a large scale seemed to be important factors for these accidents.

The massive SARS-CoV-2 vaccination campaigns have mitigated the burden of the pandemic; however, along with vaccination, the risk for healthcare personnel followed. Large-scale vaccination required the support of young medical and nursing personnel in training (7). our data suggest that this could be related to increased occupational accidents.

Furthermore, issues, such as healthcare coverage and legal responsibility for HP and employers during these brigades, are not clear-cut.

Since vaccination seems to be the strongest public health measure for reducing the morbidity and mortality of SARS-CoV-2 infection, there will continue to be a need to vaccinate most of the population. Large-scale brigades will continue in many regions for an indefinite period, with a high demand placed on HP to deliver these vaccinations (8).

Although in the United States, the Needle stick Safety and Prevention Act requires OSHA to emphasize the use of engineering controls, specifically, devices with sharp injury prevention features for a higher degree of protection,^1^ these devices are rarely available in other countries where legislation and work safety is not a primary concern. Injuries from hypodermic needles used for SARS-CoV-2 vaccines are already the most prevalent type associated with NI. This fact, coupled with a high patient output, extended working hours, and stress, can further increase NI risk. On-the-field workplace safety, adequate health training, and raising awareness in HPs on preventing NI are fundamental.

Stress burden during the COVID-19 crisis has impacted NSI, which was demonstrated in a study conducted by Stojic et al. (9) where the burden placed on the health system by the pandemic resulted in a higher incidence of NI despite a lower total number of patients treated at the hospital dedicated to COVID-19 in addition to unprepared medical professionals who had to participate which may also have contributed to the higher rate of NI. We acknowledge that we could not calculate individual rates for each HP by doses applied and the vaccine brand associated with these accidents since that information was not clear. In the current study, most of the accidents occurred in medical personnel in training highlighting a lack of experience. We believe that the data expressed here will aid in the implementation of preventive measures, especially for medical personnel in training.

## Data availability statement

The raw data supporting the conclusions of this article will be made available by the authors, without undue reservation.

## Ethics statement

The studies involving human participants were reviewed and approved by Comité de Ética del Hospital Universitario Dr. José Eleuterio González. Written informed consent for participation was not required for this study in accordance with the national legislation and the institutional requirements.

## Author contributions

JM-G gathered the information and prepared the draft. MP-O reviewed the manuscript, final version of the manuscript, and gathered information. AG-L and HO-B provided valuable information from the front line. AC-O contributed with the original idea, revised draft, and final version of the manuscript. All authors contributed to the article and approved the submitted version.

## Footnotes

^1^https://www.osha.gov/laws-regs/regulations/standardnumber/1910/1910.1030#1910.1030(d)(2)(i) (accessed August 20, 2022).
